# In rural Gambia, do adolescents have increased nutritional vulnerability compared with adults?

**DOI:** 10.1111/nyas.13587

**Published:** 2018-03-25

**Authors:** Simon M. Schoenbuchner, Sophie E. Moore, William Johnson, Mohammed Ngum, Bakary Sonko, Ann Prentice, Andrew M. Prentice, Kate A. Ward

**Affiliations:** ^1^ MRC Elsie Widdowson Laboratory Cambridge UK; ^2^ Division of Women's Health Kings College London London UK; ^3^ School of Sport, Exercise and Health Sciences Loughborough University Loughborough UK; ^4^ MRC Unit The Gambia Banjul The Gambia; ^5^ MRC Lifecourse Epidemiology Unit University of Southampton, Southampton General Hospital Southampton UK

**Keywords:** nutrition, growth, Africa, puberty, anemia, body composition

## Abstract

Adolescents may be particularly susceptible to malnutrition owing to the energy and nutrient costs of the pubertal growth spurt. Here, our aim is to compare differences in selected markers of nutritional status between adolescents and adults in rural Gambia. The Keneba Biobank collects cross‐sectional data and samples for all consenting individuals resident in the West Kiang region of the Gambia. For this study, participants between the ages of 10 and 40 years were selected (*n* = 4201, females 2447). Height, body mass index, body composition, hemoglobin concentration, fasting glucose concentration, and blood pressure were compared using linear regression models adjusting for age, parity, season of measurement, and residence, across three age groups: early adolescent (10–14.9 years), late adolescent (15–19.9 years), and adult (20–39.9 years). Adolescents, particularly early‐adolescent girls and boys, were shorter, lighter, and leaner than adults. By late adolescence, differences were smaller, particularly in girls where, notably, the prevalence of overweight, hypertension, and impaired fasting glucose was low. Given the importance of maternal health for reproductive outcomes and intergenerational health, the results of the study, albeit with limited biomarkers available, indicate that adolescent girls are no more compromised than adult women or males from the same population.

## Introduction

In 2011, the population of adolescents aged 10–19 years in the world was 1.2 billion.^1^ In sub‐Saharan Africa, 30–35% of the population are aged 10–24 years.^2^ Adolescent growth and timing of puberty are important determinants of adult and intergenerational health and noncommunicable disease risk.^3^, ^4^ Clearly, at a time of rapid growth, the energy and nutrient requirements of an individual increase substantially. As such, adolescents may be particularly susceptible to malnutrition owing to the energy and nutrient costs of the pubertal growth spurt and, for girls, the increased demands for iron after menarche. Adolescent health and nutrition are closely linked to offspring birth outcomes and can therefore also have important long‐term (intergenerational) effects. The most recent reports of pubertal timing in Gambian girls from a rural, subsistence farming population (the same population where the current study was based) showed that the median age at menarche was 14.9 years.^5^, ^6^ In boys, puberty is delayed even more than in girls, with age at peak height velocity (APHV) an indicator of the pubertal growth spurt, being reported as 16.1 ± 0.12 years.^5^, ^7^ In comparison, the APHV in a contemporary cohort of South African adolescents was 11.4 (0.7) and 14.2 (1.1) in girls and boys, respectively;^8^ similar timings were reported in two UK birth cohorts.^9^, ^10^

The Gambia has high neonatal mortality (29.9 per 1000) and maternal mortality (706 per 100,000), as well as a high rate of adolescent pregnancy.^11^ In rural areas in 2010, 58.6% of girls were married before the age of 18 years, contributing to an adolescent birth rate of 161 per 1000 (age‐specific fertility rate, 15–19 years).^12^ The effects of adolescent pregnancy may be compounded by the relatively delayed pubertal development of Gambian children.^13^

In 2012, the Keneba Biobank was established to build on the existing Kiang West Demographic Surveillance System (KWDSS) and historical longitudinal cohorts studied in the region. The aim of the Biobank was to collect phenotypic data and biological samples and to perform some analytical tests on the population, which relies primarily on subsistence agriculture, with yield fluctuating heavily across the year. In 2013, the average life expectancy at birth in the Kiang West Longitudinal Population Study was reported as 73.5 and 65.3 years in females and males, respectively.^14^ The wet season, lasting from July to October, is a hungry period, because stored staple foods from the previous year's harvest are near depleted. At the same time, adults have an increased workload in preparation for the current year's harvest. These factors lead to reduced maternal weight gain during pregnancy and reduced birth weight.^15^ Furthermore, there are ongoing effects in later life such that children born during the wet season have increased mortality from the age of 15 years onward.^16^ Of the 36 villages in the Kiang West region, three have been the sites of longitudinal studies and demographic surveys since the 1950s. These core villages (Keneba, Manduar, and Kantong Kunda) have benefited from improved access to health care, including antenatal clinics, since 1977.^14^, ^17^

Despite wide acknowledgment of the importance of adequate nutrition status for adolescent growth, few data are available, particularly from low‐ and middle‐income countries. There is therefore a need to better understand the determinants of nutritional status among adolescents to improve their current and subsequent health and the health of their children. The aim of the current study was to compare differences in selected markers of nutritional status, height, body mass index (BMI), hemoglobin, blood pressure, and body composition between male and female adolescents and adults in rural Gambia to assess whether adolescent nutritional vulnerability exists at the population level.

## Methods

### Participants

The Keneba Biobank was established in 2012, and, by September 2015, cross‐sectional data and samples for over 9000 consenting individuals had been collected. The Biobank sampling and measurements were conducted in the field, with visits scheduled to ensure even distributions of recruitment by village and season. Biobank samples are linked to the KWDSS via the KWDSS unique identifiers (DSS), which provide information on age, parity, and family structure for every individual who has been resident in Kiang West since 2004, based on 3‐monthly surveillance of the population. DSS survey and Biobank recruitment methods and information about the database structure and data flow were detailed by Hennig *et al*.^14^

For this study, participants between the ages of 10 and 40 years were selected (*n* = 4201; females 2447). These were divided into three age groups for comparison: early adolescent (10–14.9 years), late adolescent (15.0–19.9 years), and adult (20–39.9 years). Adolescents were split into two groups as per UNICEF recommendations.^1^

Parity was obtained from the DSS. It is possible that some older children may not have been included in this count if they had not lived in the Kiang West region at any time since the initiation of the DSS in 2004. Since almost all early‐adolescent girls were nulliparous, this age group was excluded from any analysis involving parity as a predictor. Very few late‐adolescent girls had had two or more children, so comparisons between age groups were made using parity as a binary variable, comparing subjects who had had at least one child to those who had not. Direct data concerning lactation were not available as part of the information collected by the Biobank. Instead, the DSS was used to determine whether a subject had had a child within the 2 years preceding the Biobank visit and to confirm whether that child was alive at the time of the mother's Biobank visit. If both of these conditions were true, due to the very high rates of breastfeeding in this population, the mother may have been lactating. Pregnancy in the last 2 years is therefore reported in Table 1 as a marker of high rates of pregnancy and breastfeeding in this population.^18^

**Table 1:** Descriptive statistics by sex and age group^1^ (mean ± SD or %)

	Female			Male		
**Age group**	10–14.99	15–19.99	20–39.99	10–14.99	15–19.99	20–39.99
***n***	764	604	1072	879	526	342
**Age (years)**	12.5 ± 1.4	17.1 ± 1.4	30.1 ± 6.0	12.5 ± 1.4	16.9 ± 1.4	28.8 ± 6.4
**Height (cm)**	146 ± 10	160 ± 6	161 ± 6	143 ± 9	164 ± 10	174 ± 7
**HAZ**	−0.96 ± 1.03	−0.39 ± 0.90	−0.27 ± 0.93	−1.35 ± 1.07	−1.25 ± 1.13	−0.35 ± 0.93
**Stunted (HAZ < –2)**	14%	3%	2%	25%	25%	3%
**Weight (kg)**	34.6 ± 8.5	50.7 ± 8.4	56.2 ± 10.4	31.8 ± 6.4	48.2 ± 9.6	62.3 ± 8.9
**BMI (kg/m^2^)**	16.0 ± 2.2	19.8 ± 2.9	21.5 ± 3.7	15.4 ± 1.7	17.7 ± 2.1	20.6 ± 2.7
**BAZ**	−1.34 ± 1.01	−0.56 ± 0.99	−0.12 ± 1.06	−1.61 ± 1.00	−1.64 ± 1.05	−0.72 ± 1.00
**Underweight (BAZ < –1.65)**	38%	12%	6%	46%	48%	20%
**Overweight (BAZ > 1.04)**	1%	5%	14%	1%	0%	4%
**Fat‐free mass (kg)**	27.1 ± 6.0	38.6 ± 4.3	40.3 ± 4.2	26.6 ± 5.3	41.7 ± 8.2	53.9 ± 6.4
**Fat mass (kg)**	6.95 ± 2.77	11.7 ± 5.1	15.4 ± 7.6	4.65 ± 1.63	5.92 ± 2.22	7.96 ± 4.52
**Fat %**	19.9 ± 3.4	22.5 ± 6.1	26.4 ± 7.3	14.8 ± 3.3	12.2 ± 3.1	12.5 ± 5.4
**FAZ**	−0.95 ± 0.83	−0.49 ± 1.23	0.17 ± 1.19	−0.85 ± 0.90	−1.16 ± 0.97	−1.08 ± 1.59
**Low body fat (FAZ < –2.05)**	7%	10%	3%	9%	16%	25%
**High body fat (FAZ > 1.04)**	1%	9%	24%	2%	1%	9%
**Fasting glucose (mmol/L)**	4.59 ± 0.51	4.64 ± 0.60	4.51 ± 0.61	4.55 ± 0.53	4.52 ± 0.48	4.53 ± 0.59
**IFG (fasting glucose > 6.1)**	0%	1%	1%	1%	0%	1%
**Hemoglobin (g/dL)**	12.2 ± 1.2	12.1 ± 1.3	11.6 ± 1.4	12.2 ± 1.1	12.9 ± 1.3	13.9 ± 1.4
**Anemia (age‐specific cutoffs** ^*^)	29%	41%	58%	35%	53%	22%
**Moderate anemia (hemoglobin < 11)**	8%	15%	27%	10%	7%	3%
**Severe anemia (hemoglobin < 8)**	1%	1%	1%	0%	0%	0%
**Systolic BP (mmHg)**	107 ± 10	111 ± 10	111 ± 13	104 ± 9	111 ± 11	119 ± 12
**Diastolic BP (mmHg)**	63 ± 8	66 ± 8	70 ± 10	60 ± 8	62 ± 8	69 ± 9
**Hypertensive (age‐specific cutoffs** a **)**	8%	4%	5%	5%	2%	5%
**Core village resident**	20%	23%	20%	23%	29%	21%
**Wet season visit (July–October)**	33%	30%	29%	33%	35%	34%
**Parity 1+**	1%	20%	87%	0%	0%	0%
**Parity 2+**	0%	5%	76%	0%	0%	0%
**Parity 3+**	0%	0%	63%	0%	0%	0%
**Pregnant in the last 2 years**	0%	7%	52%	0%	0%	0%

HAZ, BAZ: height‐ and BMI‐for‐age Z–scores, based on the WHO references. FAZ: fat %–for‐age Z–score based on Tanita reference data.^20^ IFG, impaired fasting glucose.

a Age‐specific cutoffs were used to define mild anemia, as recommended by Ref. 18: 11.5 g/dL until age 12 years, 12.0 g/dL until age 15 years; 12.0 g/dL after age 15 years (female), and 13.0 g/dL after age 15 years (male). Moderate and severe anemia were defined using thresholds of 11 and 8 g/dL, respectively (in males and females of all ages); hypertension was defined as systolic BP > 140 or diastolic BP > 90 for participants aged 18 or over, or according to U.S. guidelines for adolescents.^22^

### Anthropometry

Weight and height were collected using standard protocols and regularly validated equipment. BMI was computed at weight (kg)/height squared (m^2^). Height and BMI were converted to Z‐scores (height‐for‐age (HAZ); BMI‐for‐age (BAZ)) using the WHO growth references.^19^ The reference centiles attain adult values at the age of 19. Since adolescent growth in the Gambia is known to be delayed relative to reference populations, the centiles for 19‐year‐olds were also applied to all participants over the age of 19, so that adult values could be compared to those of adolescents.^13^ Stunting, underweight, and overweight were defined as per WHO definitions: stunting, HAZ < –2; underweight, BAZ < –1.65 (below the fifth percentile); overweight, BAZ > 1.04 (above the 85th percentile). At the age of 19 years, the fifth percentile of BMI is close to 18 kg/m^2^, the threshold normally used to define underweight in adults, so the same cutoffs are used in all age groups.

### Body composition

Body composition was measured by bioimpedance using a Tanita BC‐418 MA Segmental Body Composition Analyser (Tanita Corporation, Amsterdam, the Netherlands). The outcome used in this study was body fat expressed as a percentage of total body mass. Fat percent–for‐age Z‐score (FAZ) was calculated using reference centiles based on a European population that was measured using the same model of bioimpedance analyzer.^20^ Low body fat was defined as FAZ < –2.05 (below the second percentile) and high body fat as FAZ > 1.04 (above the 85th percentile), as recommended.^20^

### Hemoglobin

Hemoglobin concentration was measured from fasting venous blood samples as part of a full blood count using a Boule Medical Medonic M‐series 3‐part Haematology Analyser (Boule Diagnostics AB, Sweden). Age‐specific cutoffs were used to define mild anemia, as recommended:^18^ 11.5 g/dL up to 11.99 years, 12.0 g/dL up to 14.99 years, 12.0 g/dL after age 15 years (female), and 13.0 g/dL after age 15 years (male). Moderate and severe anemia were defined using thresholds of 11 and 8 g/dL, respectively (in males and females of all ages).

### Blood pressure

Blood pressure was measured using an Omron 705‐CPII. For subjects aged 18 years or over, hypertension was defined as systolic blood pressure (SBP) ≥ 140 mmHg and/or diastolic blood pressure (DBP) ≥ 90 mmHg.^21^ For subjects under the age of 18, hypertension was defined according to U.S. guidelines for children and adolescents.^22^

### Fasting glucose

Fasting glucose was measured from a venous blood sample using a Roche Diagnostics Accu Check (London, UK). A fasting glucose concentration of 6.1 mmol/L or more was considered indicative of impaired fasting glucose (IFG).^23^

### Statistical analysis

Statistical analysis was carried out in R version 3.3.2,^24^ including the following packages: dplyr version 0.5.0 for data manipulation and ggplot2 version 2.2.1 for plotting.^25^, ^26^ Continuous outcome variables were HAZ, BAZ, FAZ, hemoglobin concentration, and SBP and DBP. These were compared across the three age groups using linear regression. Residence of the core village of Keneba, Manduar, and Kantong Kunda was included as a binary predictor. Season of measurement was also included as a binary predictor, comparing the wet (hungry) season lasting from July to October against the dry season. For female participants, the analysis was repeated twice: first, for all participants and then limited to nulliparous women. To test whether age differences depended on village of residence or season, interactions were added to the initial models. These were nonsignificant in all cases and so not reported or included. Coefficients for the age‐group terms in the unadjusted models were nearly identical to those in the models adjusted for season and village, and so only the adjusted models are included in the results. For each model, QQ plots were inspected to check for normality of the residuals.

Comparisons between the adolescent groups and adults are given in Table 2. Coefficient estimates are shown with 95% confidence intervals and are considered significant if *P* < 0.05.

**Table 2:** Comparisons between markers of nutritional status in adolescent and adult females and males from the Kiang West region of the Gambia

			Height–age Z–score	BMI–age Z–score	Percent fat–age Z–score	Hemoglobin (g/dL)	Systolic blood pressure (mmHg)	Diastolic blood pressure (mmHg)
**Female**	**All (*n* = 2440)**	**Intercept (adult)** a	−0.30 [−0.36, −0.23]*	−0.14 [−0.21, −0.07]*	0.21 [0.14, 0.29]*	11.71 [11.62, 11.80]*	111.75 [110.97, 112.53]*	70.60 [70.01, 71.20]*
		**Early adolescent**	−0.70 [−0.79, −0.61]*	−1.22 [−1.32, −1.13]*	−1.11 [−1.21, −1.00]*	0.59 [0.46, 0.71]*	−3.91 [−4.98, −2.84]*	−7.34 [−8.16, −6.52]*
		**Late adolescent**	−0.13 [−0.22, −0.03]*	−0.44 [−0.55, −0.34]*	−0.65 [−0.76, −0.54]*	0.47 [0.33, 0.60]*	−0.48 [−1.63, 0.68]	−4.55 [−5.42, −3.67]*
		**Core village**	−0.00 [−0.09, 0.09]	0.12 [0.02, 0.23]*	0.06 [−0.04, 0.17]	0.12 [−0.01, 0.25]	−0.42 [−1.55, 0.71]	−0.56 [−1.43, 0.30]
		**Wet season**	0.11 [0.03, 0.19]*	−0.01 [−0.10, 0.08]	−0.20 [−0.30, −0.10]*	−0.31 [−0.43, −0.20]*	−1.12 [−2.11, −0.12]*	−1.34 [−2.10, −0.58]*
	**Nulliparous (*n* = 1382)**	**Intercept (adult)** a	−0.47 [−0.64, −0.31]*	−0.18 [−0.35, −0.01]*	0.08 [−0.10, 0.25]	11.87 [11.66, 12.09]*	114.23 [112.52, 115.95]*	71.26 [69.88, 72.64]*
		**Early adolescent**	−0.54 [−0.72, −0.36]*	−1.18 [−1.36, −1.00]*	−0.94 [−1.13, −0.76]*	0.41 [0.18, 0.64]*	−6.37 [−8.18, −4.56]*	−7.90 [−9.36, −6.45]*
		**Late adolescent**	0.06 [−0.13, 0.24]	−0.49 [−0.68, −0.30]*	−0.55 [−0.75, −0.36]*	0.34 [0.10, 0.58]*	−3.20 [−5.09, −1.31]*	−5.37 [−6.89, −3.85]*
		**Core village**	0.01 [−0.12, 0.14]	0.00 [−0.13, 0.13]	−0.05 [−0.19, 0.08]	0.09 [−0.08, 0.27]	−0.44 [−1.76, 0.88]	−0.45 [−1.51, 0.61]
		**Wet season**	0.13 [0.02, 0.25]*	0.02 [−0.09, 0.14]	−0.23 [−0.35, −0.12]*	−0.26 [−0.41, −0.11]*	−1.40 [−2.55, −0.25]*	−1.79 [−2.71, −0.86]*
**Male (*n* = 1747)**		**Intercept (adult)** a	−0.36 [−0.48, −0.24]*	−0.71 [−0.82, −0.59]*	−0.96 [−1.08, −0.84]*	14.04 [13.89, 14.18]*	119.52 [118.36, 120.69]*	69.89 [68.96, 70.82]*
		**Early adolescent**	−1.00 [−1.13, −0.87]*	−0.89 [−1.02, −0.75]*	0.24 [0.10, 0.38]*	−1.75 [−1.91, −1.59]*	−14.59 [−15.88, −13.31]*	−8.78 [−9.81, −7.75]*
		**Late adolescent**	−0.91 [−1.06, −0.76]*	−0.93 [−1.07, −0.79]*	−0.08 [−0.23, 0.07]	−1.04 [−1.22, −0.87]*	−7.73 [−9.14, −6.33]*	−6.40 [−7.52, −5.28]*
		**Core village**	0.03 [−0.09, 0.15]	0.14 [0.03, 0.26]*	0.17 [0.05, 0.29]*	0.11 [−0.03, 0.25]	−0.49 [−1.63, 0.64]	−0.09 [−0.99, 0.82]
		**Wet season**	0.02 [−0.09, 0.13]	−0.15 [−0.26, −0.05]*	−0.49 [−0.60, −0.38]*	−0.43 [−0.56, −0.30]*	−2.54 [−3.56, −1.52]*	−2.90 [−3.71, −2.08]*

note: Data are presented as coefficients from linear regression models, adults being the referent group; for sex interactions, females are the referent group. Data shown are mean estimates with 95% confidence intervals, ^*^ *P* < 0.05. Models were fitted for all female participants, nulliparous female participants, and male participants separately.

a The intercept refers to the average outcome in adults not residing in core villages in the dry season.

## Results

**Figure 1:**
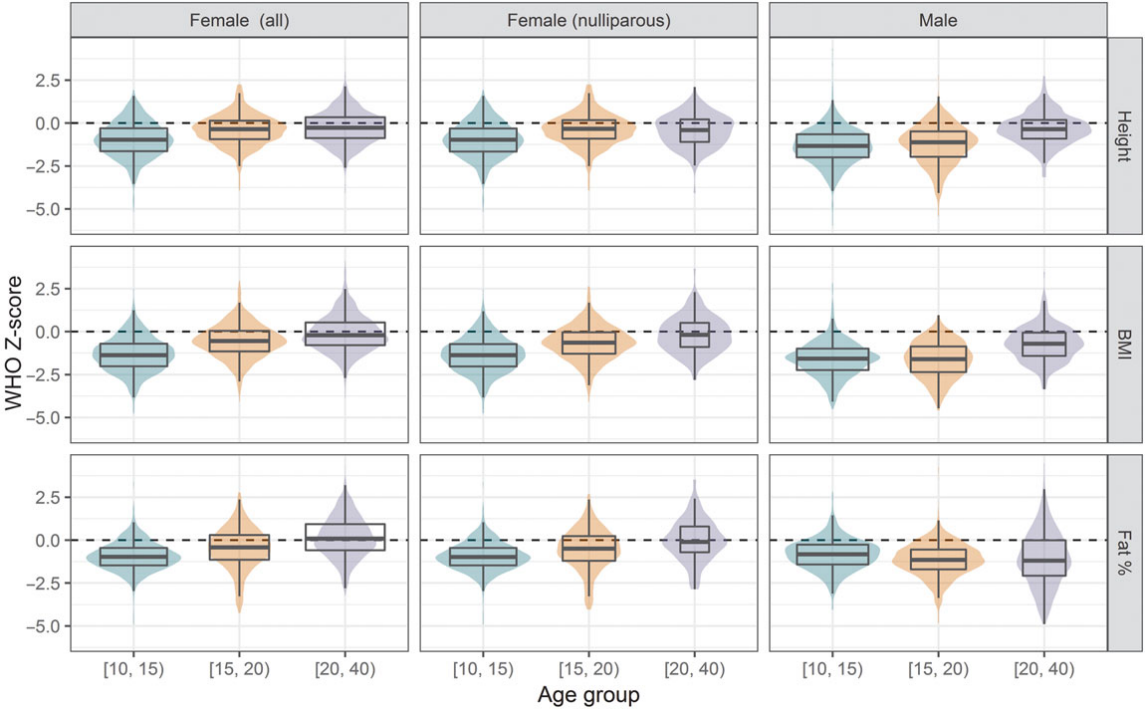
Height‐, BMI‐, and fat %‐for‐age Z‐scores by age group and sex. Box plots showing the median (thick horizontal line), interquartile range (height of box), and sample size (width of box), superimposed on violin plots showing the distribution. The dashed horizontal line indicates a Z‐score of zero, representing the median in the international reference data.

Descriptive statistics for each age group are shown in Table 1. Continuous variables are summarized as means ± standard deviations and categorical variables as percentages. The distributions of the anthropometric outcome variables across age groups are illustrated in Figure 1. Between 20% and 29% of respondents in each age group were from the core villages of Keneba, Manduar, and Kantong Kunda, and there was an even distribution across months of the year for data collection across the age groups. Many late‐adolescent girls (20%) had had at least one child, and a majority of adult women (63%) had had at least three children. For 52% of adult women, the most recent child had been born within the last 2 years, suggesting that they might have been lactating at the time of the visit.^18^

Stunting was common among adolescent boys (25%) and among early‐adolescent girls (14%). Underweight was common among adolescent boys (early 46% and late 48%), adult men (20%), and adolescent girls (early 38% and late 12%), but adult women were more likely to be overweight (14%). Low body fat was common among late‐adolescent boys (16%) and girls (10%) and among adult men (25%), whereas high body fat was common among adult women (24%). Anemia was common in all age groups and both sexes (from 22% in adult men to 58% in adult women), but this was rarely severe. Hypertension and IFG were rare.

### Female (all)

Adolescent girls in both age groups were shorter and had lower BMI and fat percent than adult women, relative to the reference populations. They also had lower blood pressure (lower SBP in early adolescence and lower DBP in both age groups) but higher hemoglobin concentration. Residency in the core villages was associated with a higher BMI. Fat percent, hemoglobin, and blood pressure were lower during the wet season, but height was greater.

### Female (nulliparous)

When considering only nulliparous women, results remained very similar to those in the complete female group. Exceptions were that the difference in fat percent was no longer significant in early adolescence. SBP was significantly lower in late adolescence, and there was no longer an effect of core village residence on BMI.

### Male

Adolescent boys were shorter and had lower BMI than adult men, relative to the reference population. Fat percent was lower in early adolescence but not in late adolescence. Hemoglobin concentration and blood pressure were lower in both adolescent groups than in adults. Core village residence was associated with increased BMI and fat percent. During the wet season, BMI, fat percent, hemoglobin, and blood pressure were all lower than during the dry season.

### Pooled females and males

Sex differences in adult HAZ were not significant, but men had lower BMI, lower fat percent, higher hemoglobin concentration, and higher SBP than women. The differences among age groups were significantly different between the sexes for all outcomes except DBP. There were also significant sex differences in the effects of core village residence on fat percent and in the effect of season of measurement on BMI and fat percent.

## Discussion

In a population‐based study of over 4000 individuals from a rural, subsistence farming population susceptible to malnutrition, this work has shown, that toward the end of growth and in early–mid‐adulthood, there is low prevalence of stunting, overweight, and obesity and in risk factors for cardiometabolic disease and anemia. Males remain lighter and leaner than females, but overall there were no significant differences between females and males for the markers of health assessed.

There were greater differences with age in BAZ and FAZ among boys than among girls, which is consistent with the more delayed pubertal development of boys compared with girls. This is in contrast to previous reports of nutritional vulnerability in adolescent girls but is possible owing to an increased sensitivity of males to environmental challenges.^1^, ^5^, ^7^, ^8^, ^27^ However, owing to known delays in pubertal development in this population, these results cannot necessarily be interpreted as a signal of nutritional vulnerability.^13^ Growth references are created according to chronological age, which is the simplest, globally consistent assessment of an individual's age. However, it is well recognized that chronological age is limited as a reflection of maturity, particularly when delayed or precocious puberty occurs due to an underlying chronic disease or, as is the case in the current population, due to environmental constraint. As such, it is difficult to determine whether a low HAZ or BAZ is due to a physiological deficit in growth or to delayed puberty, and, in turn, the subsequent impact on final height or body composition. Previous work from the Gambia indicated that the longer growth period and later puberty in the Kiang West population allow some catching up of height growth.^13^ Consistent with this, the results of the current cross‐sectional study suggest that delayed puberty is the likely reason for the observations of group differences in growth. This is best seen for HAZ, where Z‐scores are almost zero in the adult group, indicating the longer and slower growth period in this population does not, at the population level, impair final adult height. Similar to girls’ BAZ, FAZ Z‐scores are close to zero by the end of growth. To confirm these observations, longitudinal studies and/or more direct assessment of pubertal stage or maturity, such as bone aging or Tanner staging, are required.^8^, ^28^ Regardless, the impact of later or slower pubertal growth on increased risk of future disease has been well described in higher income populations, showing later pubertal growth to be associated with higher risk of cardiovascular and musculoskeletal disease.^9^, ^10^, ^29^ In low‐ and middle‐income countries (LMICs), less is known about the impact of pubertal timing on linear and somatic growth during adolescence or on final height, weight, and future health outcomes.^30^ In the COHORTS collaboration, a greater BMI gain during childhood and adolescence was associated with poorer markers of cardiometabolic health. Similarly, recent data from the Vellore cohort in India showed that greater height and weight gain relative to height were associated with increased risk of a poor cardiovascular disease profile in adulthood.^31^

One surprising finding was the impact of village of residence within this rural community on both BAZ and FAZ, with higher values observed in residents of the three core villages. This finding probably reflects a combination of the closer proximity to the MRC Keneba clinic and the impact of the MRC on the economy in this area (through direct employment and also indirectly).^17^ The observation draws parallels with recent work from the urban Gambian population, where the odds of being stunted were increased if a child's parents had been born in a rural region.^27^

In addition to the importance of adolescent growth for health of the individual, there are also impacts for intergenerational health. Younger maternal age (≤19 years) is a risk factor for a range of adverse pregnancy outcomes, including low birthweight, preterm birth, and childhood stunting at 2 years of age.^32^ As adolescence is a period of nutritional vulnerability, it is easy to assume that these adverse outcomes are a consequence of nutritional constraint during this time. However, we have published elsewhere that the literature on the nutritionally mediated pathways underpinning the links between young maternal age and poor intergenerational and long‐term health is sparse.^4^ The data presented here suggest that, even in a rural sub‐Saharan African context, where seasonally driven food insecurity creates a high risk of undernutrition in childhood,^33^ adolescent girls are no more susceptible to nutritional vulnerabilities than adolescent boys or older females and males, at least for the limited number of nutritional status biomarkers assessed. However, as this analysis was cross‐sectional in nature and did not include a cohort of pregnant adolescent girls, we are unable to comment on how pregnancy would affect nutritional status.

There are limited comparable data reporting population‐level trends in markers of nutritional status across females and males in this age range. A recently published review presenting data on global and regional trends in the nutritional status of young people highlighted that “while national‐level data for children under 5 have been largely collected, over‐ and undernutrition data for adolescents are mostly unavailable and tend to have smaller sample sizes.”^3^ Indeed, in this same review and using recent estimates from the WHO's Global School‐Based Health Survey of underweight prevalence by WHO region and for the age groups 13–15 years, 16–17 years, and 13–17 years, only 16 of 59 counties listed had comparable data across the early‐to‐late adolescent period.^3^ Where data were available, there was limited evidence of differential vulnerability by age group, at least for underweight (<–2 SD from median for BMI). However, for the previously discussed reasons, there is a need to extend the use of HAZ, WAZ, and BAZ to include other biomarkers in order to gain a full and in‐depth reflection of the underlying nutritional vulnerabilities in the population.

In the current study, for measures of nutritional status other than height and BMI, fewer and less consistent associations were observed for trends between age groups. Of note, our data highlight that particularly within the late‐adolescent group, females were in relatively good nutritional and metabolic health with no impaired glucose control, little hypertension, and low rates of moderate and severe anemia. Hemoglobin concentration was higher in adult men than in adolescent boys, but this relationship was reversed among women and girls. Lower hemoglobin concentrations of adult women may be indicative of insufficient recovery between multiple pregnancies, but the difference was still evident when the analysis was limited to nulliparous females. Blood pressure increased with age from early to late adolescence and from late adolescence to adulthood. This increase was greater among males than among females, though none of these increases indicated high rates of hypertension in the populations. Blood pressure was lower during the wet (hungry) season than during the dry season, and there was no difference between the core villages and other villages. No differences were found for fasting glucose concentrations.

The strengths of the current study include the relatively large size of the cohort, with contemporary data, and focused on a population susceptible to nutritional challenges. Limitations have been discussed but briefly include the limited availability of biomarkers, lack of detailed socioeconomic status, lack of assessment of maturational status, and the fact that cross‐sectional data do not allow underlying etiology to be determined. Furthermore, the reliance on published reference data and cutoffs, often generated from contrasting populations, and the use of different references and cutoffs between age groups may introduce some inherent errors into the data as presented.

In conclusion, in older adolescent females and males from rural Gambia, there is little evidence to suggest that, in comparison with adults from the same population, prevalence of anemia, stunting, overweight and obesity differs. Overall, these data indicate good cardiometabolic health in this population. High rates of anemia across the ages indicate nutritional vulnerability at a population level. The data from girls and boys in early adolescence, although indicative of higher levels of undernutrition, are limited in its interpretation owing to the aforementioned issues caused by the known delay in puberty in the Kiang West Population, which can create an artifactual exaggeration of differences from reference populations.^5^, ^6^, ^13^ These results may not be applicable to populations at different stages of nutrition, social, and economic transition. Moving forward, work should include direct assessment of a maturational marker or pubertal status and nutritional status, dietary intake, and markers of socioeconomic status to further define the concept of nutritional vulnerability in adolescents. Such measurements would include assessment of micronutrient status for nutrients such as zinc, iron, sodium, vitamin D, folate, and vitamin A.^34^ Clearly, adolescence remains an important period of an individual's life course and is an important determinant of adult and intergenerational health. It is important to extend the work to other settings and to continue to monitor the current population, which is in nutritional, social, and economic transition, to fully understand the impact of adolescence in future noncommunicable disease risk.

## Competing interests

The authors declare no competing interests.
